# Fertility Outcome after Operative Laparoscopy versus No Treatment in Infertile Women with Minimal or Mild Endometriosis

**Published:** 2012-03-20

**Authors:** Ashraf Moini, Laleh Bahar, Mansour Ashrafinia, Bita Eslami, Reihaneh Hosseini, Narges Ashrafinia

**Affiliations:** 1 Department of Obstetrics and Gynecology, Arash Women’s Hospital, Tehran University of Medical Sciences, Tehran, Iran; 2 Department of Endocrinology and Female Infertility, Reproductive Biomedicine Research Center, Royan Institute for Reproductive Biomedicine, ACECR, Tehran, Iran; 3 Vali-e-Asr Reproductive Health Research, Tehran University of Medical Sciences, Tehran, Iran; 4 Tehran University of Medical Sciences, Tehran, Iran

**Keywords:** Clinical Trial, Infertility, Laparoscopy, Endometriosis, Pregnancy

## Abstract

**Background:**

Endometriosis is the presence of endometrial glands and stroma in sites other than the uterine cavity, which is associated with infertility. The objective of this study was to evaluate the effect of laparoscopic surgical treatment on clinical pregnancy in infertile women with minimal or mild endometriosis.

**Materials and Methods:**

This clinical trial study was performed in infertile women who were referred to the gynecological clinic between April 2008 and March 2009. After confirmation of minimal or mild endometriosis by diagnostic laparoscopy, patients were randomly assigned into two groups using consecutively numbered, opaque sealed envelops. The first group consisted of women who only underwent diagnostic laparoscopy (no treatment) before randomization. The second group of patients underwent operative laparoscopies. T-test and chi-square test were used when appropriate. A p-value less than 0.05 was considered statistically significant.

**Results:**

Analysis with 38 patients in each group showed characteristics such as age, body mass index (BMI), and duration of infertility were statistically similar in both groups. At 9 months follow- up, 9 (24%) women who had operative laparoscopies became pregnant compared with 7 (18%) women in the diagnostic laparoscopy group. The pregnancy rate showed no statistically significant difference between both groups (p=0.49). No complications were reported in either group.

**Conclusion:**

The present study suggested that laparoscopic surgical treatment was not superior to diagnostic laparoscopy in pregnancy occurrence in infertile women with minimal and mild endometriosis. (IRCT Number: IRCT201012311952N2).

## Introduction

Endometriosis is the presence of endometrial glands or stroma in sites other than reproductive lives. It is associated with symptoms such as pelvic pain, dysmenorrheal, painful sexual intercourse and infertility (1). It is estimated that there is a 10% prevalence of endometriosis in the general population (2).

Evidence has shown that endometriosis is a dynamic benign disease where the majority of women do not improve if untreated (3, 4).

Improvement of fertility ability in women with endometriosis has been investigated by various medical (5) or surgical methods (6) or the combination of both. Medical therapy has often been unsuccessful and the effects of surgical methods are not completely distinct.

There are some controversies about the treatment of minimal and mild endometriosis and its effect on fertility. The result of one study has shown the fecundity rate of patients with unexplained infertility and women with mild or minimal endometriosis did not statistically differ and was not significant. That study, however, showed a trend toward decreased fecundity in women with endometriosis (7).

A study by Marcoux et al. (6) used diagnostic laparoscopy to evaluate the effects of resection or ablation of visible endometriosis in women with minimal or mild endometriosis. The cumulative rate of pregnancy was statistically higher in the surgery group (30.7%) when compared with diagnostic laparoscopy group (17.7%, p=0.0006).

In comparison, in Italy, a similar randomized control trial by Parazzini in 2000 compared diagnosis alone versus treatment of stage I/II endometriosis. The researchers did not find any significant differences in birth rates (20% vs. 22%) between the two groups. Their study did not support the hypothesis that ablation of endometriotic lesions markedly improved fertility rates (8).

A recent meta-analysis in 2010 also demonstrated the advantage of laparoscopic surgery when compared to diagnostic laparoscopy in clinical pregnancy rates (9).

Because of the controversies regarding laparoscopic treatment, it was suggested to perform an additional investigation in a different population. Therefore, the objective of our study was to evaluate the effect of laparoscopic surgical treatment on clinical pregnancy in infertile Iranian women with minimal or mild endometriosis.

## Materials and Methods

This clinical trial study was performed in infertile women who were referred to the Gynecological Outpatient Clinic of Arash Hospital between April 2008 and March 2009.

Inclusion criteria were: age between 20-32 years, unexplained infertility more than 1 year, normal semen analysis, normal ovulatory cycles (menstrual interval 24-35 days and biphasic basal temperature), normal hormonal assay [thyroid stimulating hormone (TSH), follicle-stimulating hormone (FSH), prolactin (PRL)] and normal hysterosalpingography.

Women who met the following criteria were excluded from our study: surgical history for infertility or endometriosis, oophorectomy, salpingectomy, history of pelvic inflammatory disease (PID) and those who received any treatment for endometriosis during the previous 3 months.

The Ethics Institutional Review Board of Tehran University of Medical Sciences approved the study and informed consent was obtained from all participants after counseling regarding the potential risks of laparoscopy.

Laparoscopy was performed under general anesthesia by the standard approach. Triple-puncture laparoscopy was performed, with a 10-mm operating laparoscope inserted through an umbilical port and two 5-mm sheaths inserted in the lower quadrants lateral to the inferior epigastric vessels. Pneumoperitoneum was established and maintained using insufflators capable of delivering carbon dioxide (CO2) at a pressure of 8-10 mmHg. Endometriosis was diagnosed as one or more scattered, superficial endometrial implants on the pelvic peritoneum, no more than 5 mm in diameter on one or both ovaries and uterine serosa, without depth involvement and adhesion formation.they are the criteria of mild to moderate endometriosis. Disease stage was defined according to the Revised American Fertility Society (R-AFS) classification (10). Scores from 1 to 5 were considered as stage 1 (minimal), whereas scores from 6 to 15 were stage 2 (mild). If there was a higher stage of endometriosis, or if adhesion formation distorted the natural anatomy or there was any occlusion in one or both tubes during laparoscopy, the patient was excluded from the study.

After confirmation of minimal or mild endometriosis by diagnostic laparoscopy in all patients, eligible women were randomly assigned to surgery or diagnostic laparoscopy only using consecutively numbered, opaque sealed envelops by a surgical assistant while the patient was still anesthetized. Patients were unaware of their treatment assignment.

Operative laparoscopy patients underwent ablation to remove any visible endometrial implants. During surgery, the surgeon chose the type of procedure. The removal was done by bipolar cauterization (Wolf Co. Germany). In difficult anatomic positions, implants were cauterized with the fulguration method without complete resection. Diagnostic laparoscopy patients were simply followed and controlled.

No medical treatment for endometriosis or infertility was prescribed during the follow-up period; if patients used any hormonal medications they were excluded from the study. The primary outcome of the present study was normal intrauterine pregnancy occurrence during 9 months of follow-up.

If human chorionic gonadotropin (hCG) was positive, sonography would be performed for confirmation of normal intrauterine pregnancy. Clinical pregnancy was defined by the visualization of an embryo with cardiac activity at 6-7 weeks of pregnancy.

The secondary outcome was complications during or post-surgery, such as: intestinal injury, slight tear of the tubal serosa, vascular trauma, infection of wounds, hematomas, and urinary tract infection.

Based on the results of Marcoux et al. (6), we estimated the rate of pregnancy in the surgical group as 15% and the diagnostic group, 35%. Thus, we calculated that 73 patients would be required in each group to detect differences in pregnancy rate with a power of 90% and α=0.05 by Epi Info (www.cdc.gov/epiinfo/).

Statistical analysis was performed by SPSS software (version 13). The quantitative data was displayed as mean ± standard deviation and the qualitative data as numbers with percentages. T-test, chi-square test or Fisher’s exact test were used when appropriate. A p-value less than 0.05 was considered statistically significant.

**Fig 1:**
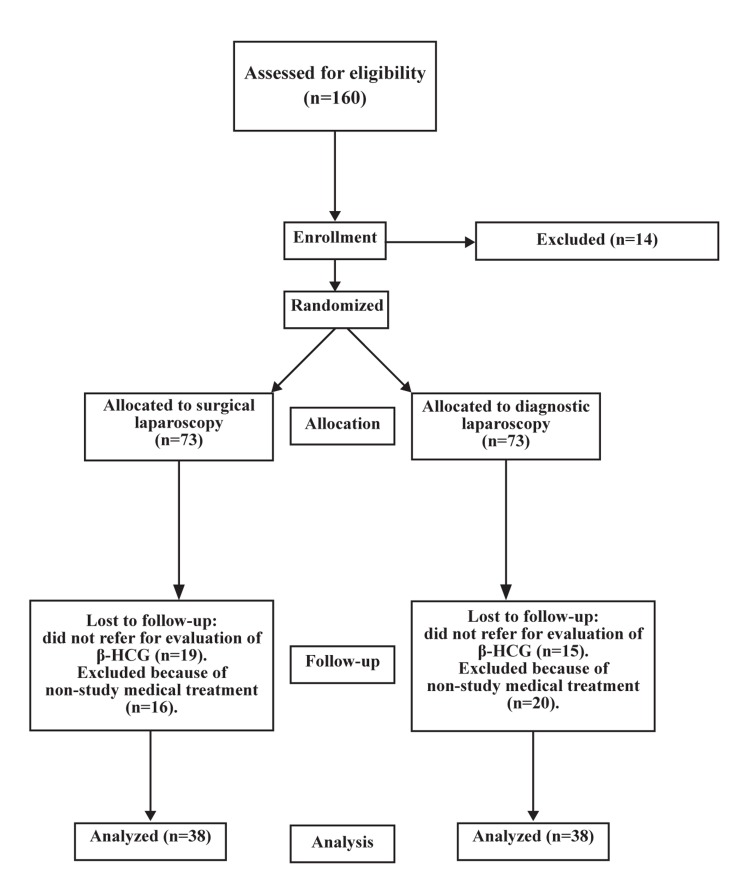
Flow-chart of patient participation.

## Results

There were 73 patients who met the inclusion criteria and randomly assigned to 2 groups. Figure 1 shows the flowchart of patient participation in our study. Some patients withdrew from the study follow-up or took medical treatment during the follow-up period. Therefore, for analysis there were 38 patients in each group who continued participation.

Age, body mass index (BMI) and duration of infertility were statistically similar in both groups (p>0.05). As expected, the anesthesia time was significantly higher in the operative group (82 ± 22.7 minutes) than the diagnostic group (60.9 ± 12.4 minutes; p<0.001; Table 1).

At 9 months follow-up, there were 9 (24%) women in the operative laparoscopy group who became pregnant compared with 7 of the 38 (18%) women in the diagnostic laparoscopy group. The pregnancy rate was not statistically significant between both groups (p=0.49; Table 2).

**Table 1:** Total characteristics in both groups


Variables	Operative laparoscopy(n=38)	Diagnostic laparoscopy(n=38)	P value

**Age (years)**	27 .8 ± 3.3	27.7 ± 3.1	0.82
**BMI (kg/m^2^) **	22.6 ± 2.4	22.4 ± 2.3	0.8
**Duration of infertility (years)**	3.3 ± 1.6	2.8 ± 1.1	0.9
**Anesthesia time (minutes)**	82 ± 22.7	60.9 ± 12.4	<0.001
**Staging of endometriosis Minimal**	20 (52.63%)	22 (57.89%)	1
**Mild**	18 (47.37%)	16 (42.11%)	0.36


P value refers to t test and chi-square test.

**Table 2:** The pregnancy rate in two groups after 9 months follow-up


	Operative laparoscopy	Diagnostic laparoscopy	P value

**Pregnancy rate**	9/38 (23.7%)	7/38 (18.4%)	0.49


P value refers to chi-square test.

Overall, no surgical complications were encountered in both groups and all patients were discharged from the hospital one day after laparoscopy.

## Discussion

Because the clinical course of minimal endometriosis is not predictable, any benefits from specific medications remain uncertain. There is no evidence of medical treatment modalities altering the clinical course of minimal and mild endometriosis.

The exact mechanisms and association between endometriosis and infertility is unknown. Oocyte development or early embryogenesis and reduced endometrial receptivity (11) may be responsible for infertility in endometriosis. Certainly, the overall prevalence of endometriosis appears greater among infertile than fertile women (12).

Some studies have manifested the improvement or relief of pelvic pain as an important symptom in endometriosis and intra- or post-operative complications with different surgical laparoscopic techniques (13-15).

In this study the main outcome was only pregnancy rate; we did not score pelvic pain in this population.

Data from the present study suggested that laparoscopic surgical treatment had no statistically significant effect in pregnancy occurrence in infertile women diagnosed with minimal and mild endometriosis.

However, 2 clinical trials of surgical laparoscopy in patients with minimal and mild endometriosis had different results (6, 8). In a clinical trial in Canada, 341 patients with unexplained infertility who underwent laparoscopy and were diagnosed with minimal and mild endometriosis were randomly divided between 2 groups of surgical treatment and expected management. Patients were followed for 9 months. Out of 172 patients, 50 (29%) in the surgical group became pregnant, while 29 from 169 (17%) in the controlled group became pregnant. This study showed that surgical therapy was effective in improvement of fertility (6). On the contrary, comparison of surgical laparoscopy with diagnostic procedure during 1 year follow-up showed a 24% vs29% pregnancy rate in unexplained infertile women with minimal or mild endometriosis. Thus their result did not support the hypothesis that ablation of endometriosis lesions markedly improved fertility rates (8). Another study had shown that either no treatment or surgery was superior to medical treatment for minimal and mild endometriosis associated with infertility. For moderate and severe disease, surgery was recommended (16).

On the other hand, another study by Fuchs et al. noted a high pregnancy rate (65%) within an 8.5 month post-surgical time, of which 86.5% pregnancies resulted in deliveries (17).

Nardo et al. reported the cumulative pregnancy rate at 23.2% after laparoscopic treatment with the Helica Thermal Coagulator for minimal and mild endometriosis (13).

A systematic review in 2010 has reported that the use of laparoscopic surgery in the treatment of subfertility related to minimal and mild endometriosis may improve future fertility (9). In total, some studies have suggested that surgery for the treatment of early stage endometriosis is probably effective (18,19).

Therefore, whether surgical treatment is more effective than medical or no treatment in minimal and mild endometriosis is still a matter of scientific debate. On the other hand, the gynecologist's ability to determine fertility prognosis based on disease staging is limited.

This study was the first academic evaluation of infertile women with mild and minimal endometriosis in Iran. As shown, the results have been reported by another study. However, there is some controversy regarding minimal and mild endometriosis and its effects on fertility. This should be evaluated more in different societies.

The limitation of the present study was its low power due to small sample size. Long time follow-up caused increased patient loss because many patients with infertility desire to get pregnant as soon as possible and cannot refuse to take any medical treatment during the follow-up period. Thus, they were excluded from this study because of medical treatment for endometriosis or infertility. Therefore, further studies with larger sample sizes would be required to evaluate the exact effect of operative laparoscopy on fertility outcome.

## Conclusion

This study suggests that although pregnancy results are not significant in both groups of patients with mild to moderate endometriosis, the results are the same as other studies. For better evaluation, future studies with larger numbers of patients should be conducted.
